# Flaxseed Oil (*Linum usitatissimum*) Prevents Cognitive and Motor Damage in Rats with Hyperammonemia

**DOI:** 10.3390/nu15214550

**Published:** 2023-10-27

**Authors:** Marcos F. Ocaña-Sánchez, Gabriel A. Soto-Ojeda, Yolanda Cocotle-Ronzón, Cesar Soria-Fregozo, Alberto Sánchez-Medina, Rosa V. García-Rodríguez, Juan F. Rodríguez-Landa, Erick J. Corro-Méndez, Minerva Hernández-Lozano

**Affiliations:** 1 Programa de Doctorado en Ciencias Biomédicas, Centro de Investigaciones Biomédicas, Universidad Veracruzana, Xalapa 91190, Mexico; 2 Facultad de Química Farmacéutica Biológica, Universidad Veracruzana, Xalapa 91090, Mexico; gsoto@uv.mx (G.A.S.-O.); ycocotle@uv.mx (Y.C.-R.); 3 Laboratorio Ciencias Biomédicas/área Histología y Psicobiología, Departamento de Ciencias de la Tierra y de la Vida, Centro Universitario de los Lagos, Universidad de Guadalajara, Lagos de Moreno 47460, Mexico; cesar.soria@academicos.udg.mx; 4 Instituto de Química Aplicada, Universidad Veracruzana, Xalapa 91190, Mexico; albsanchez@uv.mx (A.S.-M.); rosagarcia02@uv.mx (R.V.G.-R.); 5 Instituto de Neuroetología, Univerisdad Veracruzana, Xalapa 91190, Mexico; juarodriguez@uv.mx; 6 Facultad de Ciencias Biológicas y Agropecuarias, Universidad Veracruzana, Amatlán 94945, Mexico; ecorro@uv.mx

**Keywords:** hyperammonemia, flaxseed oil, hepatic encephalopathy

## Abstract

Hyperammonemia is characterized by the excessive accumulation of ammonia in the body as a result of the loss of liver detoxification, leading to the development of hepatic encephalopathy (HE). These metabolic alterations carry cognitive and motor deficits and cause neuronal damage, with no effective treatment at present. In this study, we aimed to evaluate the effect of two subacute oral administrations of flaxseed oil (0.26 and 0.52 mL/kg) on short- and long-term memory, visuospatial memory, locomotor activity, motor coordination, and the neuronal morphology of the prefrontal cortex (PFC) via tests on Wistar rats with hyperammonemia. The goal was to identify its role in the regulation of cerebral edema, without liver damage causing cerebral failure. In contrast with an ammonium-rich diet, flaxseed oil and normal foods did not cause cognitive impairment or motor alterations, as evidenced in the short-term and visuospatial memory tests. Furthermore, the flaxseed oil treatment maintained a regular neuronal morphology of the prefrontal cortex, which represents a neuroprotective effect. We conclude that the oral administration of flaxseed oil prevents cognitive and motor impairments as well as neuronal alterations in rats with hyperammonemia, which supports the potential use of this oil to ameliorate the changes that occur in hepatic encephalopathy.

## 1. Introduction

Ammonia is an intermediate metabolite for protein and amino acid synthesis, and it contributes to an acid–base balance in organisms. This metabolite has the ability to cross the blood–brain barrier, and in the brain, it participates in the transformation from glutamine to glutamate. Similarly, it becomes a product of protein metabolism degradation, which is expelled from the body in the form of urea synthesized in the liver [1].

When a liver injury occurs, regardless of its origin, high levels of ammonia are present in the blood as well as in the central nervous system, causing different effects, such as high intracellular osmolarity, which causes edema and the loss of astrocytes, releasing inflammatory cytokines, such as tumor necrosis factor alpha (TNFα), interleukins 1 and 6, and interferon. In the remaining astrocytes, the inhibition of α-ketoglutarate dehydrogenase and the depletion of carbon skeletons occur, consequently paralyzing the Krebs cycle [2].

Therefore, the decreased expression of glutamate receptors in astrocytes induces increased glutamate concentrations, leading to seizures, increased cerebral blood flow, the loss of effective cerebral autoregulation, and the development of cerebral edema and intracranial hypertension [3]. This is how the loss of liver detoxification can lead to hyperammonemia and cause hepatic encephalopathy (HE), a disease characterized by cognitive and motor impairments, particularly involving the glutamatergic neurotransmission pathway [4].

This disease comprises a clinical picture of various neuropsychological disorders, with cognitive, emotional, personality, motor activity, memory, and consciousness manifestations, and patients can even fall into a coma. It can episodically or continuously appear and is reversible in most cases [5]. There is compelling evidence of prefrontal cortex alterations associated with hepatic damage attributed to the accumulation of ammonia crossing the blood–brain barrier. These neuroanatomical changes represent the underlying correlate of cognitive impairments observed in both clinical cases and animal models [6,7,8].

The current treatment for this disorder involves the removal of ammonia in the blood; hence, antibiotics and non-absorbable disaccharides are used, mainly rifaximin and lactulose [9]. However, there are certain factors that contradict the use of this antibiotic, such as its high cost, and long-term use by cirrhotic patients is a cause for concern. Furthermore, both lactulose and rifaximin are used to decrease the concentration of ammonia in the body, but they do not have an impact on the impaired liver; thus, we need to develop alternative treatments [10]. On the other hand, flaxseed oil is derived from *Linum usitatissimum* seeds, and it contains various compounds including caffeic acid, p-coumaric acid, ferulic acid, secoisolarisiresinol diglucoside, and secoisolariciresinol. Additionally, this oil is abundant in unsaturated fatty acids, such as oleic, linoleic, palmitic, and stearic acids, with linoleic acid being the most prevalent, constituting nearly half of the total fatty acids. Moreover, flaxseed oil is rich in polyphenolic compounds, which contribute to its significant antioxidant potential [11]. Linoleic acid has been shown to have cytoprotective effects in different models [12,13,14,15] and in clinical trials, as it acts as an antioxidant and inhibitor of proinflammatory cytokines [16,17,18,19,20,21].

Flaxseed is commonly available for human consumption in three different forms: whole, ground, and oil flaxseed. Their stability, bioactive components, and pharmacokinetic parameters depend on the way they are consumed. The alpha-linolenic acid (ALA) in whole seeds withstands temperatures of up to 350 °C and maintains oxidative stability, but when the seed’s outer shell is crushed, this stability decreases. This oil should be stored in opaque glass containers, be refrigerated, and have an unrefined form to prevent oxidation and preserve its bioactive properties [22]. Flaxseed oil can contain up to 50% ALA when it is cold-pressed, and several studies have examined the bioavailability of flaxseed oil using both high doses of 20 g and low doses ranging from 1.2 to 5.2 g per day. These studies have shown that the consumption of flaxseed oil increases the plasma levels of ALA. However, some studies have concluded that higher doses close to 20 g are necessary to elevate the EPA levels in both plasma and erythrocytes [23].

Therefore, in this study, we aimed to evaluate the cognitive and motor effects of orally administrating flaxseed oil to model rats with hyperammonemia who ate an ammonium-containing diet to identify its role in regulating cerebral edema, without hepatic damage causing cerebral failure.

## 2. Materials and Methods

### 2.1. Ethics

We carried out experiments on animals in accordance with the guidelines of the Care and Use of Laboratory Animals (National Research Council, 2011) and Norma Oficial Mexicana para el Cuidado y Uso de Animales de Laboratorio (NOM-ZOO-062, 1999). Additionally, the 3Rs principles (reduce, refine, replace) were taken into consideration in preclinical research [24].

### 2.2. Flaxseed Oil

Flaxseed oil was obtained from the capsules of the dietary supplement Flax Seed Oil 1000^®^ from GNC (General Nutrition Center, Xalapa, Mexico). Prior to administration, the oil from each capsule was extracted using a removable insulin syringe needle. The capsule’s content was analyzed using mass spectrometry, and a total of 43% alpha-linolenic acid was determined per capsule.

### 2.3. Animals

Male Wistar rats weighing 150–200 g were used in this study. They were kept in a vivarium in the Faculty of Pharmaceutical and Biological Chemistry of the Universidad Veracruzana in translucent acrylic boxes (45 × 30 × 10 cm) with a 12 h light/dark cycle (lights on at 7:00 a.m.), with free access to water and food (4 per box).

### 2.4. Induction of Hyperammonemia

An ammonium-containing diet was prepared from dry rodent food mixed with 20% *w*/*w* ammonium acetate. For eight weeks, 28 rats had access to an ammonium-containing diet ad libitum [25].

### 2.5. Experimental Groups and Treatments

On the fifth week, the 28 rats were divided into four different treatments (*n* = 7 each): (A) hyperammonemia control (HaC); (B) ibuprofen 30 mg/kg/day ip (HaIbu); (C) flaxseed oil ig 0.26 mL/kg/day (HaFOD1); and (D) flaxseed oil ig 0.52 mL/kg/day (HaFOD2). An additional group was used as a control, which was administered mineral oil ig and fed a standard diet for the duration of the experiment (control group). All the treatments were administered during the final 3 weeks of the experiment. Once the treatments were completed, behavioral tests were conducted, followed by blood and brain tissue collection. These two doses of flaxseed oil were based on the Mantzioris et al. 2000 study and adjusted after analyzing the composition of alpha-linolenic acid using mass spectrophotometry with a nutritional supplement (Flax Seed Oil 1000^®^, Xalapa, Mexico). Additionally, Rodrigo et al. (2010) justified the use of intraperitoneal ibuprofen at a dose of 30 mg/kg to reduce neuroinflammation induced by hyperammonemia in an animal model of encephalopathy without liver damage, as well as in the bile duct ligation model [26,27,28].

### 2.6. Behavioral Test Battery

After completing all the treatments, the animals of each group were individually evaluated using a behavioral test battery, including open field, spontaneous object recognition, Barnes maze, and Rotarod tests.

#### 2.6.1. Open Field Test

The open field test was used to evaluate locomotion, which consisted of an opaque acrylic box measuring 81 × 81 cm with a height of 40 cm, whose floor was divided into 64 squares of 10 × 10 cm. First, a pre-test session (5 min) was conducted, and after 24 h, the number of squares crossed by the animal after 5 min was evaluated, as well as the frequency of rearing activity and the time spent grooming.

#### 2.6.2. Spontaneous Object Recognition Test

To evaluate short- and long-term memory, the spontaneous object recognition test (SOR) was used, in which short-term memory was measured one hour after the end of the training phase, and long-term memory was measured 24 h later. Once exploration duration data were obtained, the percentage of time spent exploring the novel object during each test was calculated relative to the total exploration time spent focused on either of the two objects.

#### 2.6.3. Barnes Maze

The Barnes maze was used to evaluate visuospatial memory. Ten trials were conducted per subject in the acquisition phase, and 24 h after the trials were completed, the memory test was performed. The escape latency (in the case of the acquisition phase, the area under the curve (AUC) was used) was determined. Likewise, the number of errors made during the acquisition phase were checked (primary errors); this refers to the instances where the subject inserted their head into incorrect holes before reaching the goal or successfully escaping.

#### 2.6.4. Rotarod

Motor coordination was evaluated using the Rotarod test. An automated 4-line Rotarod RS system (LE 8300, LSI Letica, Panlab Scientic Instruments, Barcelona, Spain) for rats was used, in which the animals were placed in a 7 cm diameter cylinder, after habituating for 3 min for two consecutive days with the apparatus turned off. On the third day, the test was performed with a speed from 4 to 40 rpm for 300 s, during which the latency until the first fall post-treatment administration was measured, with a maximum cut-off point of 600 s [29].

### 2.7. Histology

After conducting the behavioral experiments, the animals were euthanized via decapitation after being anesthetized with Xylazine (Cheminova de Mexico SA de CV, CDMX, Mexico) (2 mg/kg). Their brains were extracted and stored in 4% formaldehyde solution in phosphate buffer until further use. They were refrigerated at 4 °C for 24 h before being transferred to phosphate buffer pH 7.4 for 1 h before sectioning. Coronal sections of 30 μm thickness were obtained using a Leica VT 1200 S vibratome (Leica Microsystems CMS GmbH, Wetzlar, Germany) and subsequently stained using the hematoxylin-eosin technique. From each brain, 50 sections were obtained, and an average number of neurons was calculated. Neuronal counting was performed via the optical microscopy of layer III of the prefrontal cortex; these images were acquired using Leica Application Suite in conjunction (Leica Microsystems AG, Heerbrugg, Schweiz) with a Leica DMi1 microscope (Leica Biosystems Nussloch GmbH, Nussloch, Germany). Then, 500 microns were measured inward, creating a rectangular area of 250 × 500 microns for analysis. The presence of degenerated cells was determined by counting neurons exhibiting pyknosis, karyorrhexis, karyolysis, cytoplasmic eosinophilia, or the loss of hematoxylin affinity, as well as dark, festooned, or edematous neurons.

### 2.8. Statistical Analysis

All data were analyzed using the R software version 4.3.1 through one-way ANOVA for independent samples. Tests that did not meet the normality criteria were analyzed using non-parametric statistics. All statistical tests were two-sided, and a post hoc Tukey test was performed when *p* ≤ 0.05. The results were plotted using GraphPad Prism 6 software (Prism Mac 6.0h). Data are presented as the mean and standard error of the mean.

## 3. Results

### 3.1. Locomotor Activity and Motor Coordination

Table 1 shows that hyperammonemic animals tend to locomote more, although this was not statistically significant with the different experimental treatments (F_(4,30)_ = 2.673, *p* = 0.051, NS). On the other hand, the hyperammonemic group reared more (F_(4,30)_ = 4.959, *p* = 0.003) and spent less time grooming (F_(4,30)_ = 5.391, *p* = 0.002).

**Figure 1:**
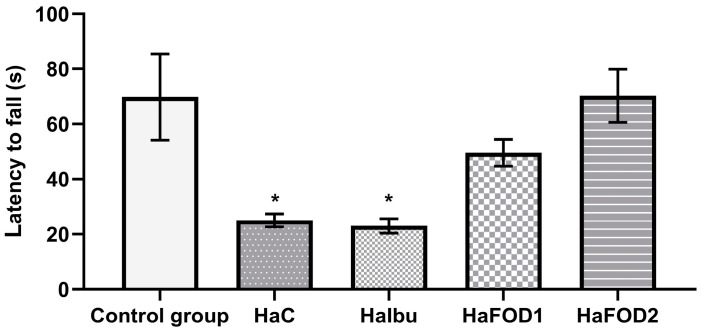
Time taken to fall off Rotarod in the motor coordination test. * *p* < 0.05 vs. all other treatments. HaC, hyperammonemia control; HaIbu, ibuprofen 30 mg/kg/day ip; HaFOD1, flaxseed oil ig 0.26 mL/kg/day; HaFOD2, flaxseed oil ig 0.52 mL/kg/day.

Figure 1 demonstrates that the hyperammonemic group (HaC) took less time to fall off the Rotarod (F_(4,30)_ = 7.144, *p* = 0.0003), an effect that was prevented with both doses of flaxseed oil treatment (HaFOD1 and HaFOD2) but not with the ibuprofen prototype (HaIbu).

### 3.2. Short- and Long-Term Memory

**Figure 2:**
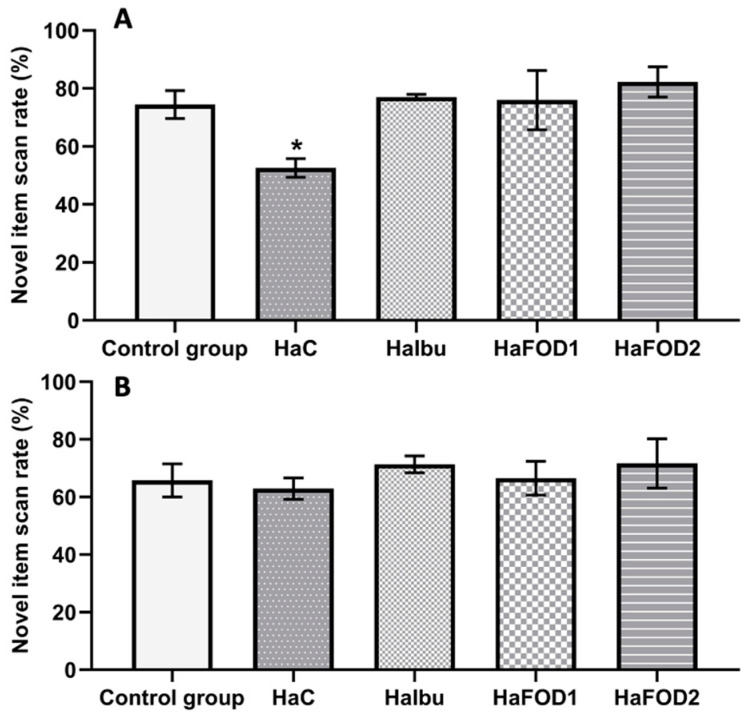
Spontaneous object recognition test. (**A**) Percentage of time spent focused on the novel object in the short-term memory test. (**B**) Percentage of time spent focused on the novel object in the long-term memory test. * *p* < 0.05 vs. all other treatments. HaC, hyperammonemia control; HaIbu, ibuprofen 30 mg/kg/day ip; HaFOD1, flaxseed oil ig 0.26 mL/kg/day; HaFOD2, flaxseed oil ig 0.52 mL/kg/day.

Figure 2A shows that during the 1 h post-familiarization test, the hyperammonemic group did not distinguish between the familiar and novel objects, spending equal amounts of time exploring both objects. In contrast, the flaxseed oil and ibuprofen groups showed a preference for examining the novel object (F_(4,30)_ = 3.977, *p* = 0.01). However, in the test conducted 24 h after the familiarization phase, no significant differences in the exploration of objects were observed in any of the groups (Figure 2B) (F_(4,30)_ = 0.44, *p* = 0.779).

### 3.3. Visuospatial Memory

**Figure 3:**
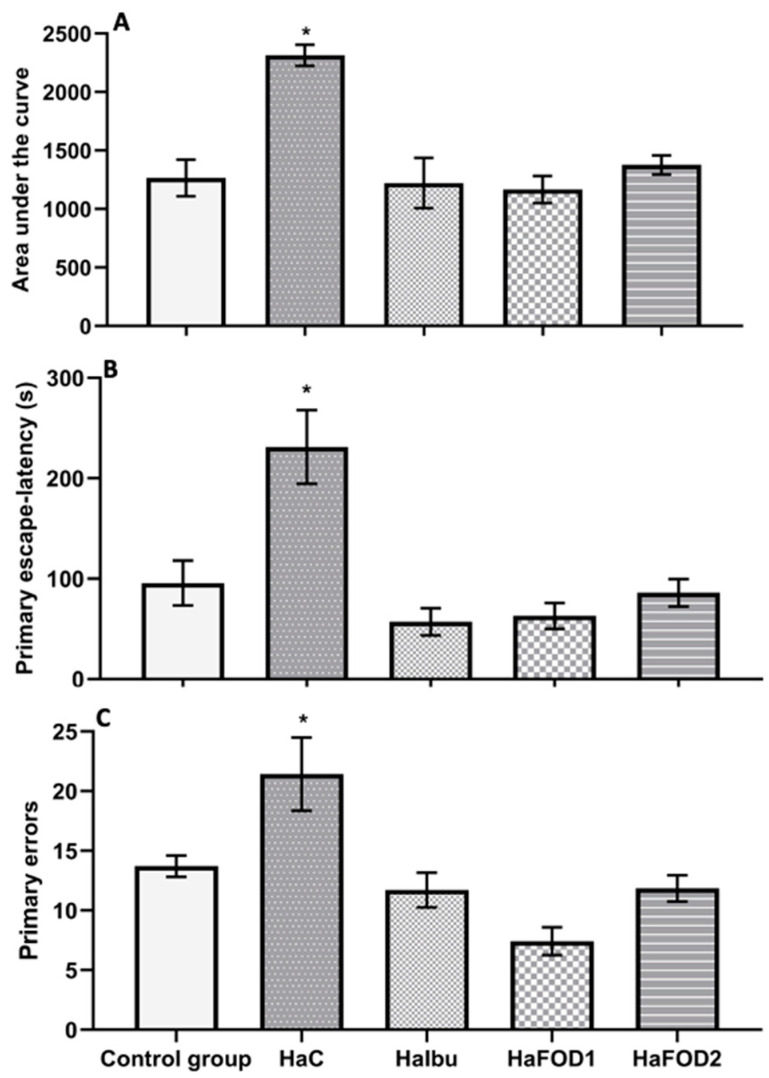
Behavioral tests to assess memory in the Barnes maze visuospatial memory test. (**A**) Area under the curve obtained from the 10 trials performed during the acquisition phase. (**B**) Time taken to find the goal in the 24 h test after the trials. (**C**) Number of errors made during the acquisition phase. * *p* < 0.05 vs. all other treatments. HaC, hyperammonemia control; HaIbu, ibuprofen 30 mg/kg/day ip; HaFOD1, flaxseed oil ig 0.26 mL/kg/day; HaFOD2, flaxseed oil ig 0.52 mL/kg/day.

Figure 3A illustrates that the hyperammonemic control group had a higher area under the curve (AUC) than all the other groups did (F_(4,30_) = 11.552, *p* = 8.527 × 10^−6^), indicating that they spent more time locating the goal during the trials. Similarly, in the 24 h test (Figure 3B), the hyperammonemia group spent more time finding the goal than the other groups did (F_(4,30)_ = 10.72, *p* = 1.62 × 10^−5^). However, the groups treated with both doses of flaxseed oil and ibuprofen showed a reduction in the time spent finding the goal during both the acquisition phase and the 24 h test (F_(4,30)_ = 10.72, *p* = 1.62 × 10^−5^). For the number of errors made before reaching the goal, the hyperammonemic group performed more errors than the other groups did (Figure 3C) (F_(4,30)_ = 22.31, *p* = 0.001).

### 3.4. Neuronal Morphology of the Prefrontal Cortex

**Figure 4:**
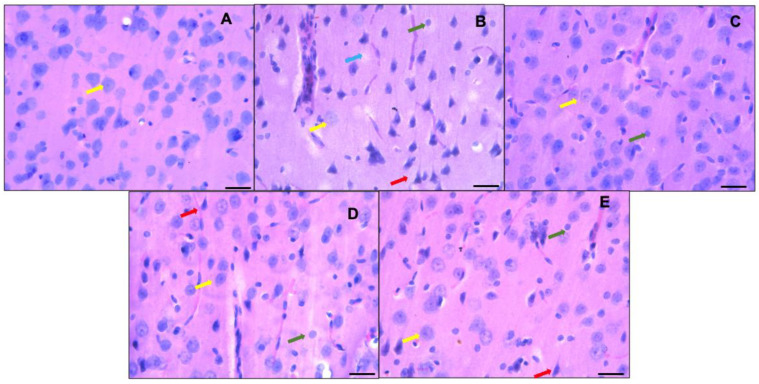
Representative micrographs showing part of the prefrontal cortex (third layer). Control group fed a standard diet ((**A**) = control group) versus groups fed ammonium-containing diet ((**B**) = control; (**C**) = ibuprofen; (**D**,**E**) = flaxseed oils D1 and D2, respectively). Normal morphologies are indicated by yellow arrows; neurons with scant cytoplasm; discretely basophilic, prominent nuclei with dispersed chromatin; and well-defined eccentric nucleoli are shown. Hyperchromatic degenerated neurons (dark neurons) are marked with red arrows. Green arrows indicate the presence of festooned-type neurons, and the blue arrow represents neurons that lose their affinity for hematoxylin or ghost neurons. Hematoxylin-eosin staining, 40×. Scale bar: 50 μm.

Figure 4 shows the histological analysis of the prefrontal cortex of the control group (animals fed a standard diet—A), where neurons with a normal morphology can be observed; the normal neurons are neurons with a small cytoplasm; discretely basophilic, prominent nuclei with dispersed chromatin; and well-defined eccentric nucleoli (indicated by yellow arrows in the images). On the other hand, the sections of the prefrontal cortices of the hyperammonemic animal control group fed an ammonium-containing diet (B) showed many dark neurons (red arrow), as well as festooned bodies (green arrow) and neuron cell bodies that lost their affinity for hematoxylin (blue arrow). In the group treated with ibuprofen (C) and in the flaxseed oil groups given doses of 0.26 mL/kg/day (D) and 0.52 mL/kg/day (E), dark neurons were still present, in addition to neurons with a normal morphology.

**Figure 5:**
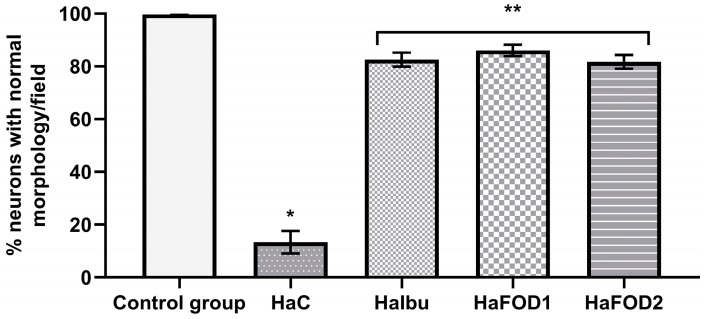
Percentage of neurons with normal neuronal morphology in the third layer of the prefrontal cortex. * *p* < 0.05 vs. all other treatments, ** *p* < 0.05 vs. vehicle. HaC, hyperammonemia control; HaIbu, ibuprofen 30 mg/kg/day ip; HaFOD1, flaxseed oil ig 0.26 mL/kg/day; HaFOD2, flaxseed oil ig 0.52 mL/kg/day.

As seen in Figure 5, the control group displayed the highest percentage of morphologically normal neurons, while the hyperammonemic control group had fewer of this type of neuron (F_(4,30)_ = 156.23, *p* < 0.001). The other hyperammonemic groups treated with ibuprofen and flaxseed oil suffered less neuronal damage and had more normal neurons than the hyperammonemic control group did. However, their neuron count was still not as high as that of the control group fed a standard diet (F_(4,30)_ = 156.23, *p* < 0.001).

## 4. Discussion

This study aimed to investigate the potential neuroprotective and behavioral effects of flaxseed oil in a hyperammonemia model induced by an ammonia-containing diet in Wistar rats. The study demonstrated that the flaxseed oil and ibuprofen treatments improved their short-term memory, visuospatial memory, locomotor activity, and motor coordination and preserved the normal neuronal morphology of the prefrontal cortex. The study involved an eight-week ammonia-containing diet regimen, with treatments of flaxseed oil and ibuprofen administered during the final three weeks.

An open field test (OFT) and a Rotarod test were utilized to evaluate locomotor activity and motor coordination. The OFT was also used to assess affective behaviors, such as the time spent grooming and rearing. Hyperammonemia leads to motor coordination impairments and anxious-like behaviors, and increased activity in animals may be due to neurological damage [30]. During the OFT, there were no changes in locomotor activity, but there was an increase in the frequency of vertical behavior and a decrease in grooming time, which were reversed by both doses of flaxseed oil. Additionally, the treatment led to an increase in the time spent on the Rotarod, indicating improved motor coordination. Hyperammonemia induces neuroinflammation, which, in turn, diminishes neurogenesis and increases cellular apoptosis in the brain. These processes contribute to the development of anxiety-like behaviors, as observed in this study [31]. On the other hand, the effects of flaxseed oil may be attributed to the anti-inflammatory properties of its primary component, alpha-linolenic acid [32]. This could potentially facilitate the restoration of neurological damage and ameliorate the motor alterations caused by excessive ammonia.

The prefrontal cortex (PFC) is involved in cognitive processing that supports executive functions such as problem solving, memory, and learning. Hepatic encephalopathy causes neuronal damage characterized by a decrease in the number of healthy neurons and the appearance of Alzheimer type II astrocytes. These characteristics are associated with cognitive and motor function impairments resulting from a cerebral edema, leading to typical symptomatic patterns [33]. Some authors have reported similar observations in animal models of hepatic encephalopathy, both in models of hyperammonemia with or without liver failure, where the number of neurons decreased in areas such as the hippocampus, cerebellum, and prefrontal cortex. Although hematoxylin and eosin staining is not a technique that shows whether neuronal tissue damage is due to apoptosis or necrosis, it has been used to presumptively identify neuronal tissue damage. This technique enables the evaluation of morphological alterations, while other techniques, such as qRT-PCR, Western blot, and immunohistochemical staining, can confirm necrosis or apoptosis. This will be taken into consideration for future studies [34,35,36].

This study shows that the control group fed a standard diet exhibited a normal neuronal morphology, without alterations, and with a cell count that follows a consistent pattern. However, the subjects of the hyperammonemic control group fed an ammonium-containing diet begin to exhibit an atypical neuronal morphology as well as a tendency toward having fewer neurons per field, which could lead to the cognitive impairment observed in the subjects. The treatments with ibuprofen and both doses of flaxseed oil restored the neuronal morphology of the subjects. The potential neurological effect of flaxseed oil is associated with its high content of alpha-linolenic acid, which has been demonstrated in several studies to be neuroprotective [12,15,37,38,39]. This acid interferes with the production of NO and the protein/mRNA expressions of iNOS, COX-2, TNF-α, and IL-6, preventing neuro-oxidation and neuroinflammation, thereby maintaining the number of neurons and the neuronal morphology of the cerebral cortices of experimental animals [38,39].

Therefore, we used the Barnes maze test (BMT) and spontaneous object recognition (SOR) test to evaluate different types of memory. The BMT is used to assess visuospatial memory, while SOR tests short- and long-term memory. In this study, the control group had a lower escape latency in both the BMT and 24 h test. In contrast, the group fed an ammonium-containing diet took more time to escape during the 10 trials and 24 h test, indicating a cognitive impairment. Interestingly, there were no differences in exploring both objects in the SOR test, suggesting a lack of discrimination between the two. This is consistent with the idea that high levels of ammonia can cause brain toxicity and cognitive damage in patients with HE and other animal models.

Ibuprofen exerts an anti-inflammatory effect by crossing the BBB and inhibiting the production of cyclooxygenases 1 and 2 (COX-1 and COX-2), thus reducing the production of prostaglandins, thromboxanes, and leukotrienes. This explains its observed effect on cerebral edema. On the other hand, although the mechanism of action of flaxseed involved in the present study was not identified, it is possible to suggest that oral administration provides alpha-linolenic acid (ALA), which can undergo β-oxidation in the liver to be transformed into docosahexaenoic acid (DHA) and cross the blood–brain barrier (BBB). Moreover, ALA can also cross the BBB with the assistance of transporter proteins. In turn, DHA inhibits the production of inflammatory cytokines, thereby reducing edema.

On the other hand, a diet rich in omega-3 fatty acids, particularly linolenic acid, and low in omega-6 fatty acids, such as linoleic acid, has been shown to have a neuroprotective effect [40,41,42,43,44]. This effect may be due to the conversion of linolenic acid into long-chain omega-3 polyunsaturated fatty acids (EPA and DHA) in vivo, although the mechanism is still debated [45,46,47]. In our study, groups fed an ammonium-containing diet and treated with flaxseed oil took longer to escape than the control group did. The effect was more pronounced in the group treated with a dose of 0.26 mL/kg/day. Additionally, flaxseed oil similarly restored the short-term memory capacity of both the treatment groups and the standard diet group. These findings suggest that a three-week treatment with flaxseed oil can restore both short-term memory and visuospatial memory in models after cognitive impairment.

In our study, we did not measure the plasma concentration of bioactive compounds, nor that of flaxseed oil, in plasma or tissue prior to the behavioral measurements. This objective was not part of our study; however, we believe that it is a natural progression in this line of research to explore pharmacokinetic parameters and variations in the bioavailability of different sources of flaxseed oil or even omega-3 itself and relate them to the biological effect. On the other hand, the two doses of flaxseed oil were based on the Mantzioris et al. 2000 study and on the analysis of the composition of alpha-linolenic acid using mass spectrophotometry with a nutritional supplement (Flax Seed Oil 1000^®^). We chose flaxseed oil due to it being one of the primary sources of omega-3 (comprising 46% of the total per capsule in this case). Additionally, it contained an appropriate proportion of omega-6 and omega-9, necessary for its biological effect. Our intention was not to evaluate isolated bioactive compounds but rather to evaluate the oil. Perhaps, in future research, it would be interesting to assess the behavior of isolated bioactive components to determine if their effects are reduced or enhanced.

## 5. Conclusions

Flaxseed oil restores motor and memory impairments, as well as neuronal morphology alterations caused by a high-ammonium diet, possibly due to its potential anti-inflammatory properties. It improves the neuronal, cognitive, and motor characteristics of rats with hyperammonemia.

## Figures and Tables

**Table 1:** Locomotor activity evaluated in the open field test.

Variable	Control Group	HaC	HaIbu	HaFOD1	HaFOD2
Square crossings	38 ± 8.68	88.6 ± 15.56	80.9 ± 11.88	83.7 ± 15.64	87.6 ± 12.13
Rearing (freq)	4.6 ± 0.92	20 ± 4.08 *	20.6 ± 3.12 *	14.6 ± 3.59	13.9 ± 1.29
Grooming (sec)	36 ± 7.13	5.7 ± 2.75 *	12.6 ± 3.89 *	39 ± 9.89	23.9 ± 4.63

* *p* < 0.05, post hoc Tukey. HaC, hyperammonemia control; HaIbu, ibuprofen 30 mg/kg/day ip; HaFOD1, flaxseed oil ig 0.26 mL/kg/day; HaFOD2, flaxseed oil ig 0.52 mL/kg/day.

## Data Availability

Data are contained within the article.
